# Exposure to outdoor artificial light at night is associated with a higher risk of ulcerative colitis: a prospective cohort study from the UK Biobank

**DOI:** 10.3389/fpubh.2026.1704450

**Published:** 2026-01-30

**Authors:** Jiamiao Chen, Laifu Li, Yan Ran, Zhuoya Sun, Shiwei Lu, Yan Zhuang, Lianli Wang, Yating Sun, Fei Dai

**Affiliations:** 1 Department of Gastroenterology, The Second Affiliated Hospital of Xi'an Jiaotong University, Xi'an, China; 2 Shaanxi Province Key Laboratory of Gastrointestinal Motility Disorders, Xi'an, China

**Keywords:** artificial light at night, inflammatory bowel disease, cohort study, Cox proportional hazard model, UK Biobank

## Abstract

**Background:**

Inflammatory bowel disease (IBD) is a chronic inflammatory condition of the gastrointestinal tract, and environmental factors are believed to play an important role in its pathogenesis. Exposure to outdoor artificial light at night (ALAN) has been linked to the globally increasing incidence and prevalence of several diseases; however, its relationship with IBD remains unclear. We aimed to estimate the long-term risk of IBD associated with outdoor ALAN exposure in a large-scale prospective cohort.

**Methods:**

We conducted a large-scale prospective cohort study using the UK Biobank. Outdoor ALAN exposure data were obtained from satellite datasets. The primary outcome was incident IBD. Cox proportional hazards regression was used to examine the association between outdoor ALAN and the incidence risk of IBD, respectively. The non-linear association was further explored using restricted cubic spline (RCS) curves.

**Results:**

During a follow-up period of 13.71 years with 346,163 participants, 1,106 individuals were diagnosed with ulcerative colitis (UC), and 508 developed Crohn’s disease (CD). After adjusting for all covariates, outdoor ALAN exposure levels were positively associated with incident UC, and an 8% higher risk of UC [hazard ratio (HR), 1.084; 95% confidence interval (CI), 1.023–1.149; *p* < 0.001] was associated with each SD increment of outdoor ALAN exposure. The highest level of ALAN exposure was associated with a significantly increased risk of incident UC compared with the lowest level of exposure. (HR, 1.309; 95% CI: 1.12–1.529; *p* < 0.001). However, no significant association was observed between outdoor ALAN and CD incidence (HR, 1.044; 95% CI: 0.83, 1.308; *p* = 0.71). Cubic splines further indicated that outdoor ALAN was non-linearly associated with UC (*p* for non-linear = 0.0063). Additionally, sensitivity analysis revealed similar results, and subgroup analysis highlighted that the interaction between outdoor ALAN and UC was stronger in women than in men.

**Conclusion:**

Our findings provide evidence that a higher ALAN exposure is associated with an increased risk of incident UC, with a significant dose–response relationship, but not with CD. Further studies are needed to elucidate the impact of outdoor ALAN on disease pathogenesis and outcomes.

## 1 Introduction

Inflammatory bowel disease (IBD) is a chronic inflammatory disorder of the gastrointestinal tract that comprises ulcerative colitis (UC) and Crohn’s disease (CD) (1). The first case report of ulcerative colitis was published by Wilks and Moxon in 1859 (2), IBD has shown an increasing incidence and now affects over one million individuals in the US and 2.5 million in Europe. The heavy burden of these diseases exerts a major toll on patients in terms of therapy, quality of life, economic productivity, and social functioning (3), making the exploration of effective prevention and treatment essential. Although the etiology of IBD remains unknown, it is thought that many factors contribute to its increasing incidence, including genetic predisposition, gut microflora, and dietary habits (4). Beyond these individual-level factors, environmental exposures associated with westernized society also appear to play a significant role in the incidence of IBD (5).

One notable environmental exposure may be exposure to artificial light at night (ALAN) outdoors. Over the past 12 years, the global extent of outdoor ALAN has increased by almost 10% annually due to urbanization (6). Excessive exposure to ALAN profoundly disrupts the natural light cycle, leading to disturbances in the circadian rhythm system and triggering a spectrum of health issues, thereby posing a substantial public health risk (7). Despite the potential for measurement error, satellite-derived ALAN data are widely used in large-scale epidemiological studies due to their accessibility. Multiple cohort studies have demonstrated that outdoor ALAN has detrimental effects on mental disorders, metabolic diseases, and sleep disorders (8–10). These findings highlight the significance of attention to the prevention and control of the adverse health effects of outdoor ALAN.

Relatively few studies have examined the link between outdoor ALAN and IBD. Evidence from animal models indicates that Bmal1−/−mutant mice, which have a non-functional circadian clock and thus no circadian rhythms, exhibit more severe colitis and delayed healing (11). Night-shift workers, who may be exposed to higher levels of ALAN, have an increased risk of developing IBD (12). Studies have suggested that disruption of the circadian clock, which could be caused by excessive pre-sleep ALAN exposure, increases the risk of IBD and contributes to its relapse and chronic fatigue (13, 14). However, no study to date has examined the association between outdoor ALAN and IBD incidence.

To address this research gap, we aimed to evaluate the association between exposure to outdoor ALAN and IBD using a large population cohort from the UK Biobank. We hypothesized that exposure to outdoor ALAN might be associated with a higher risk of IBD.

## 2 Methods

### 2.1 Study design and participants

The population data for this study were sourced from the UK Biobank, a large-scale biomedical database with over 500,000 participants from 22 assessment centers across the UK between 2006 and 2010 (15). Participants underwent a comprehensive baseline assessment that included a range of physical measurements, detailed questionnaires on lifestyle, health, and medical history, and so on. Follow-up assessments and data collection occur periodically to update health outcomes.

### 2.2 Measurement of outdoor ALAN

The outdoor ALAN data were obtained from the nighttime lights dataset freely shared by Chen et al. This study proposes a cross-sensor nighttime lights (NTLs) data calibration scheme based on autoencoders and produces the Defense Meteorological Satellite Program Operational LineScan (DMSP-OLS) system and Suomi National Polar-Orbiting Partnership Visible Infrared Imaging Radiometer Suite (NPP-VIIRS) NTL data (16). The extended time series (2000–2018) of nighttime light data is freely accessible at https://doi.org/10.7910/DVN/YGIVCD, and the unit of ALAN is nW/cm^2^/sr. UK Biobank provides participants’ address data with a 1,000-m buffer zone. After converting the address data into latitude and longitude, these coordinates were matched with the light data map to obtain each participant’s light exposure value. We also updated the matched exposure value using the most recent residential address data available from the UK Biobank.

### 2.3 Covariates

A series of covariates was considered *a priori* based on literature reviews. Demographic characteristics included age, sex, race (white or other), education phase (college/university degree or other), an indicator of poverty, and the Townsend deprivation index (TDI). Lifestyle factors included physical activity, diet, smoking status (never, occasional, or frequent), drinking status (never, former, or current), and sleep duration. According to the International Physical Activity Questionnaire (IPAQ), physical activity levels were categorized into three groups: low, moderate, and high (17). A healthy diet was defined as at least two healthy food items from the following: fruit and vegetable intake: >4.5 pieces or servings a week; fish intake: >2 servings per week; meat intake: processed meat ≤2 servings per week; and red meat ≤5 servings per week (18). Sleep duration was divided into short (<6 h per night), normal (6 ~ 9 h per night), and long (>9 h per night). Anthropometric characteristics included the average handgrip strength (defined as the average grip strength of two hands), body mass index (BMI), and waist-to-hip ratio. Standard BMI categories were used: underweight (BMI ≤ 18.5 kg/m^2^), normal weight (BMI 18.5–24.9 kg/m^2^), overweight (BMI ≥ 25.0 kg/m^2^), and obese (BMI ≥ 30 kg/m^2^). Past medical history included psychiatric disorders, hypothyroidism, hyperthyroidism, primary hypertension, and type 2 diabetes mellitus (T2DM).

### 2.4 Outcome

**Figure 1:**
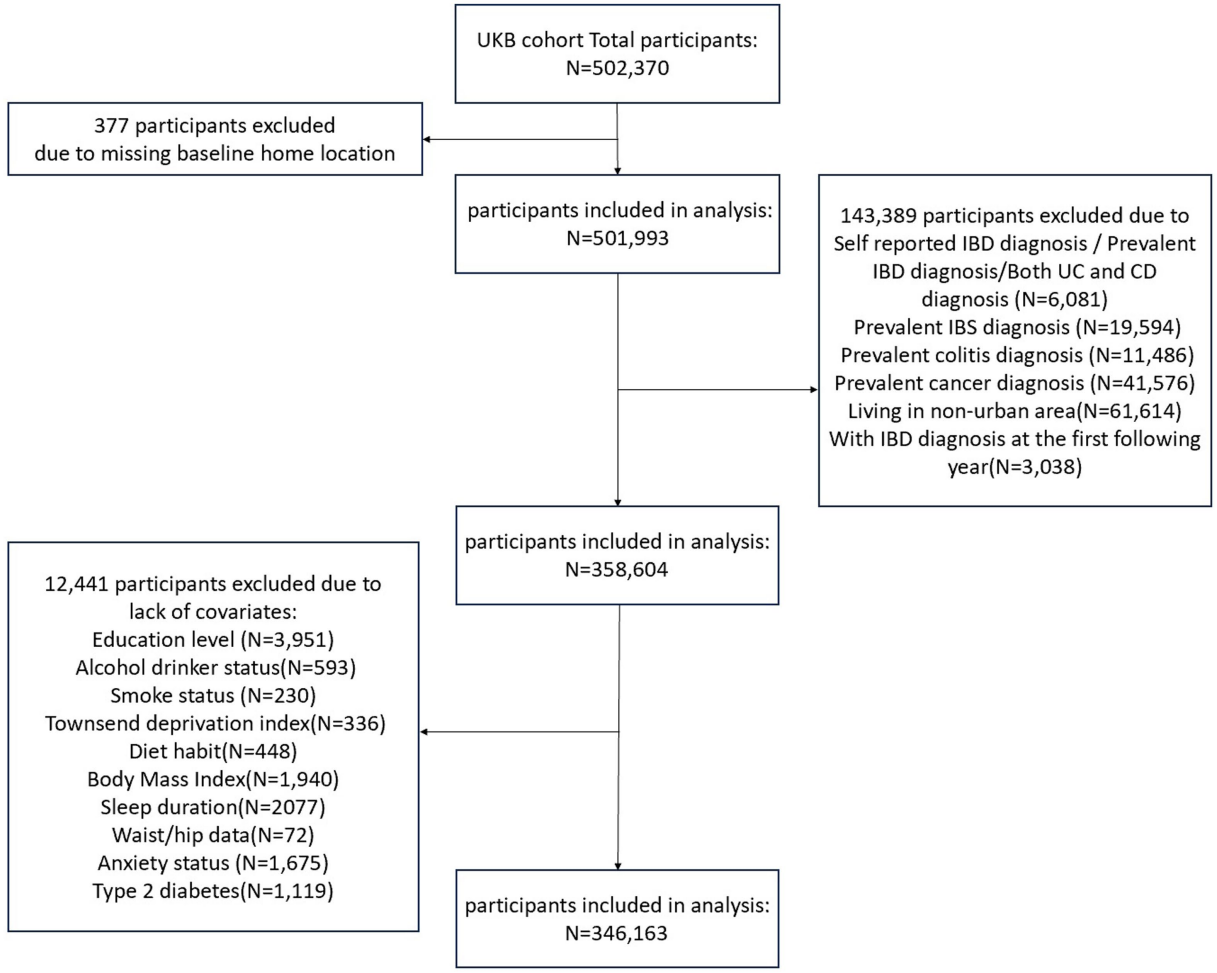
Process flowchart of inclusion and exclusion criteria. UKB, UK Biobank; IBS, irritable bowel syndrome; IBD, inflammatory bowel disease; UC, ulcerative colitis; CD, Crohn’s disease.

In the present study, incident IBD cases were identified using the 10th International Classification of Diseases codes (UC: K50; CD: K51) based on information on hospital admissions, primary care records, and self-reports. The follow-up period began on the date of the baseline assessment ended on the date of IBD diagnosis, death, loss to follow-up, or the end of the study (March 2024), whichever occurred first. The participant screening process is shown in Figure 1.

### 2.5 Statistical analysis

Baseline characteristics of the study participants were described as means [standard deviations (SDs)) for continuous variables and numbers (percentages) for categorical variables. The mean values and percentages in different groups were compared using analysis of variance (ANOVA) and *χ*^2^ tests. Cox proportional hazards regression was used to investigate the risk of incident IBD associated with outdoor ALAN exposure. The exposure was analyzed both as a continuous variable, expressed as the hazard ratio (HR) per one-SD increment, and as a categorical variable based on tertiles, with the lowest tertile serving as the reference group. To examine potential non-linear relationships, we used restricted cubic splines (RCS) with four knots to flexibly model the dose–response association between outdoor ALAN and the risks of UC and CD.

We built four models by adding covariates incrementally, including: Model 0: unadjusted; Model 1: adjusted for sex, age, ethnicity, and education level; Model 2: additionally adjusted for alcohol consumption, smoking, IPAQ, TDI, healthy diet, BMI, sleep duration, average handgrip strength, and waist-to-hip ratio; and Model 3: further adjusted by the history of disease (psychiatric disorders, hypothyroidism, hyperthyroidism, primary hypertension, and diabetes). Proportional hazard assumption was assessed and satisfied using tests based on Schoenfeld residuals in our models. Akaike Information Criterion (AIC) was used for model comparisons.

We also performed subgroup analysis with interaction tests involving stratification by age (<60 vs. ≥60 years), sex (male vs. female), diet (healthy vs. unhealthy), economic level (low vs. high), and BMI (<25 vs. ≥25 kg/m^2^) to identify the interactive factors. Several sensitivity analyses were performed to confirm the robustness of our findings: (1) repeating the main analyses after excluding participants with psychiatric disorders or sleep disorders, as these conditions may confound the association between outdoor ALAN and IBD; (2) excluding participants with IBD diagnoses within the first 3 years after recruitment; (3) further adjusting for time spent outdoors (summer and winter) and PM_2.5_ exposure.

All the above were achieved by R4.2.3^1^ and two-tailed *p* < 0.05 was considered statistically significant.

## 3 Results

### 3.1 Characteristics of participants

As listed in Table 1, our analyses included 346,163 participants and followed up for an average of 13.71 (SD: 0.95) years. Among these participants, the mean age was 56.15 (SD: 8.13) years, and males were fewer (47.8%) than females (52.2%).

**Table 1:** Baseline characteristics of the included participants.

Variables	Total (*n* = 346,163)	Non-IBD (*n* = 344,549)	UC (*n* = 1,106)	CD (*n* = 508)	*p*
ALAN (nW/cm^2^/sr) [mean (SD)]	13.33 (12.21)	13.32 (12.21)	14.53 (12.51)	13.72 (12.33)	0.002
Follow-up time (year) [mean (SD)]	13.71 (0.95)	13.74 (0.83)	8.14 (3.42)	8.13 (3.44)	<0.001
Age (year) [mean (SD)]	56.15 (8.13)	56.14 (8.13)	57.10 (8.03)	56.75 (8.28)	<0.001
Gender, *n* (%)					
Women	1,80,563 (52.2)	179,793 (52.2)	507 (45.8)	263 (51.8)	<0.001
Race, *n* (%)					
The white race	323,414 (93.4)	321,923 (93.4)	1,016 (91.9)	475 (93.5)	0.098
Degree of education, *n* (%)					
College degrees or higher	17,416 (5)	17,324 (5)	65 (5.9)	27 (5.3)	0.24
TDI (categories), *n* (%)					
Low deprivation	115,943 (33.5)	115,469 (33.5)	330 (29.8)	144 (28.3)	<0.001
Moderate deprivation	114,986 (33.2)	114,459 (33.2)	367 (33.2)	160 (31.5)	
High deprivation	115,234 (33.3)	114,621 (33.3)	409 (37)	204 (40.2)	
IPAQ, *n* (%)					
Low	52,668 (15.2)	52,397 (15.2)	190 (17.2)	81 (15.9)	0.009
Moderate	115,547 (33.4)	115,050 (33.4)	339 (30.7)	158 (31.1)	
High	114,343 (33.0)	113,831 (33.0)	352 (31.8)	160 (31.5)	
Unknown	63,605 (18.4)	63,271 (18.4)	225 (20.3)	109 (21.5)	
Smoke status, *n* (%)					
Never smoked	308,489 (89.1)	307,123 (89.1)	944 (85.4)	422 (83.1)	<0.001
Smoke regularly	27,822 (8.0)	27,631 (8.0)	127 (11.5)	64 (12.6)	
Smoke occasionally	9,852 (2.8)	9,795 (2.8)	35 (3.2)	22 (4.3)	
Alcohol status, *n* (%)					
Never	15,561 (4.5)	15,473 (4.5)	61 (5.5)	27 (5.3)	0.105
Previous	12,029 (3.5)	11,966 (3.5)	43 (3.9)	20 (3.9)	
Current	318,573 (92.0)	317,110 (92.0)	1,002 (90.6)	461 (90.7)	
BMI (categories), *n* (%)					
<18.5 kg/m^2^	1,710 (0.5)	1,701 (0.5)	6 (0.5)	3 (0.6)	<0.001
18.5–24.9 kg/m^2^	110,980 (32.1)	110,527 (32.1)	305 (27.6)	148 (29.1)	
24.9–30 kg/m^2^	147,766 (42.7)	147,088 (42.7)	479 (43.3)	199 (39.2)	
≥ 30 kg/m^2^	85,707 (24.8)	85,233 (24.7)	316 (28.6)	158 (31.1)	
Sleep duration (h), *n* (%)					
<6	118,594 (34.3)	18,729 (5.4)	73 (6.6)	140 (27.6)	0.002
6 ~ 9	117,803 (34.0)	319,936 (92.9)	1,018 (92.0)	176 (34.6)	
>9	109,766 (31.7)	5,884 (1.7)	15 (1.4)	192 (37.8)	
Grip strength (kg), *n* (%)					
Q1	121,194 (35.0)	120,653 (35.0)	374 (33.8)	167 (32.9)	0.042
Q2	120,384 (34.8)	119,845 (34.8)	376 (34.0)	163 (32.1)	
Q3	104,585 (30.2)	104,051 (30.2)	356 (32.2)	178 (35.0)	
Waist-hip-ratio, *n* (%)					
Q1	118,594 (34.3)	118,148 (34.3)	306 (27.7)	140 (27.6)	<0.001
Q2	117,803 (34.0)	117,232 (34.0)	395 (35.7)	176 (34.6)	
Q3	109,766 (31.7)	109,169 (31.7)	405 (36.6)	192 (37.8)	
Diet, *n* (%)					
Healthy	254,914 (73.6)	253,760 (73.6)	785 (71.0)	369 (72.6)	0.054
Hypothyroidism, *n* (%)					
Yes	17,060 (4.9)	16,975 (4.9)	51 (4.6)	34 (6.7)	0.568
Hyperthyroidism, *n* (%)					
Yes	3,542 (1.0)	3,519 (1.0)	15 (1.4)	8 (1.6)	0.138
T2DM, *n* (%)					
Yes	19,993 (5.8)	19,859 (5.8)	86 (7.8)	48 (9.4)	<0.001
Primary hypertension, *n* (%)					
Yes	91,531 (26.4)	309,153 (21)	342 (30.9)	170 (33.5)	<0.001
Psychiatric disorders, *n* (%)					
Yes	38,018 (11.0)	37,814 (11.0)	134 (12.1)	70 (13.8)	0.036

ALAN, artificial light at night; TDI, Townsend Deprivation Index; IPAQ, International Physical Activity Questionnaire; BMI, Body Mass Index; SD, standard deviation; T2DM, type 2 diabetes mellitus.

Throughout the follow-up, 1,106 participants were diagnosed with UC and 508 had a diagnosis of CD. Participants with IBD were more likely to have higher outdoor ALAN exposure, be older, be male, have lower economic levels, have mental disorders and primary hypertension, and engage in less physical activity (all *p* < 0.05).

### 3.2 Outdoor ALAN and risk of incident IBD

Table 2 presents the results of Cox proportional hazards model investigating the association between outdoor ALAN and IBD in the crude and three adjusted models. The crude model showed participants with the 2nd and 3rd tertiles of ALAN exposure were associated with a higher risk of incident UC (*p* for trend < 0.001). Compared with the lowest tertile, the highest tertile was associated with a 38% higher risk of incident UC (HR, 1.380; 95% CI: 1.192, 1.599; *p* < 0.001). Furthermore, continuous ALAN exposure was also significantly associated with a higher risk of UC. After adjusting for all covariates, each SD increment in ALAN exposure was associated with an 8% higher risk of UC (HR, 1.084; 95% CI: 1.023–1.149; *p* = 0.007). For CD, however, no significant association was observed between outdoor ALAN and CD incidence.

**Table 2:** The associations between exposure to outdoor ALAN and incidence of inflammatory bowel disease.

	ALAN exposure							
	Tertile 1	Tertile 2		Tertile 3		Per SD increment		*p* for trend
		*p*-value	HR 95% CI	*p*-value	HR 95%CI	*p*-value	HR 95% CI	
UC								
Model 0	Ref	0.005	1.24 (1.07–1.44)	<0.0001	1.38 (1.19–1.60)	<0.0001	1.10 (1.05–1.16)	<0.0001
Model 1	Ref	0.005	1.24 (1.07–1.44)	<0.0001	1.37 (1.18–1.59)	<0.0001	1.10 (1.04–1.16)	<0.0001
Model 2	Ref	0.013	1.21 (1.04–1.41)	<0.0001	1.31 (1.12–1.53)	0.006	1.08 (1.02–1.15)	<0.0001
Model 3	Ref	0.013	1.21 (1.04–1.41)	<0.0001	1.31 (1.12–1.53)	0.007	1.08 (1.02–1.15)	<0.0001
CD								
Model 0	Ref	0.419	1.09 (0.88–1.36)	0.162	1.16 (0.94–1.44)	0.327	1.04 (0.96–1.14)	0.327
Model 1	Ref	0.413	1.10 (0.88–1.36)	0.150	1.17 (0.95–1.45)	0.306	1.05 (0.96–1.14)	0.306
Model 2	Ref	0.661	1.05 (0.84–1.31)	0.704	1.05 (0.83–1.31)	0.938	0.99 (0.91–1.09)	0.938
Model 3	Ref	0.664	1.05 (0.84–1.31)	0.711	1.04 (0.83–1.31)	0.932	0.99 (0.91–1.10)	0.932

The results are derived from Cox hazard regression models. Model 0: unadjusted. Model 1: adjusted for age, gender, race, and educational attainment; Model 2: further adjusted for IPAQ, TDI, smoking status, alcohol consumption, BMI, diet, grip strength, and waist-to hip ratio; Model 3: further adjusted for primary hypertension, type 2 diabetes, hyperthyroidism, hypothyroidism, and mental health status. Individuals in the lowest quintile of the ALAN exposure were used as a reference; *p* < 0.05 was considered statistically significant. ALAN, artificial light at night; UC, ulcerative colitis; CD, Crohn’s disease; HR, hazard ratio; CI, confidence interval; Ref, reference group.

### 3.3 Non-linear associations between outdoor ALAN and IBD

**Figure 2:**
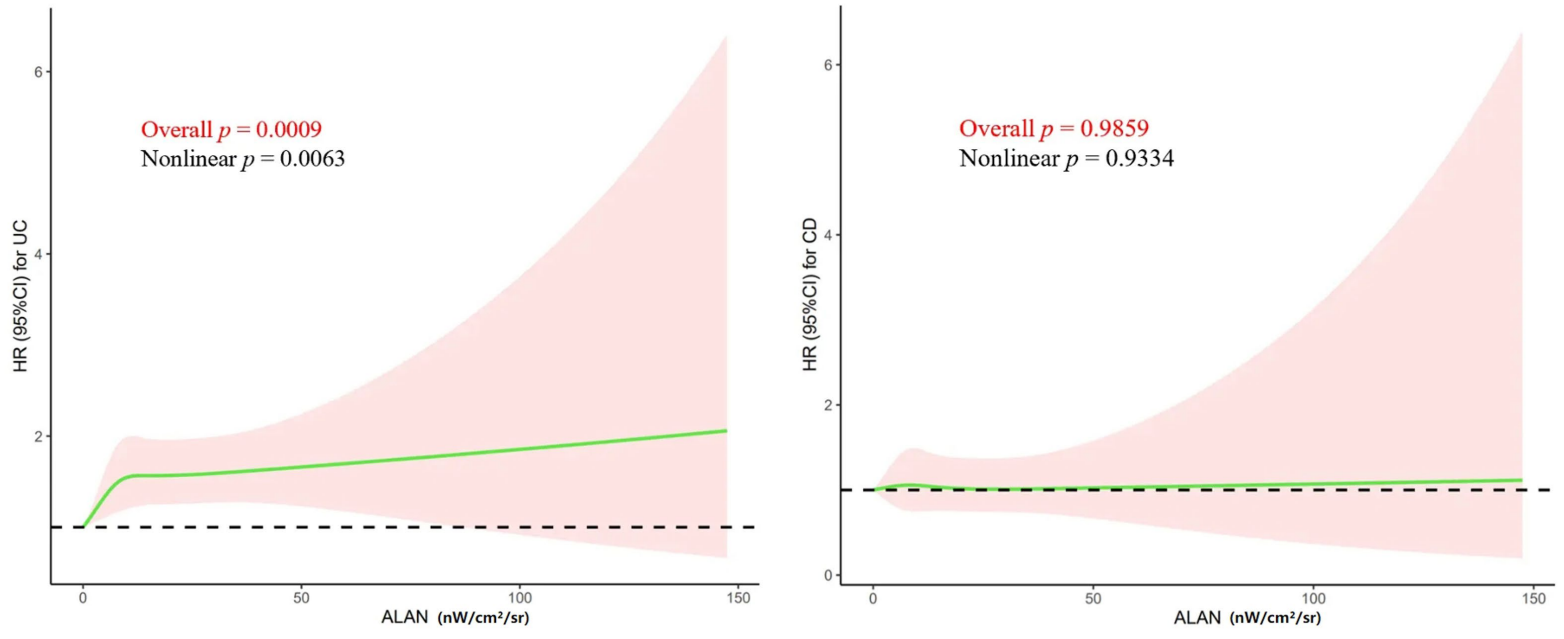
Spline curves for outdoor ALAN exposure and hazard of UC and CD incidence. Model was adjusted for age, sex, race, education attainment, IPAQ, Townsend Deprivation Index, smoking status, alcohol consumption, body mass index, diet, grip strength, waist-to-hip ratio, primary hypertension, type 2 diabetes, hyperthyroidism, hypothyroidism, and mental health status. The solid lines and shaded areas represent the central risk estimates and 95% CIs. ALAN, artificial light at night; CI, confidence interval; HR, hazard ratio; UC, ulcerative colitis; CD, Crohn’s disease. IPAQ, International Physical Activity Questionnaire.

We further- examined the shape of the exposure–response curve for the relationship between ALAN exposure and UC and CD (Figure 2). Restricted cubic splines further indicated that ALAN was non-linearly associated with UC (*p* for non-linear = 0.006). There was apparently monotonically increasing dose–response relationship for the associations between outdoor ALAN exposure and UC (*p* < 0.01), but not for CD as well (*p* = 0.98).

### 3.4 Subgroup analysis

**Figure 3:**
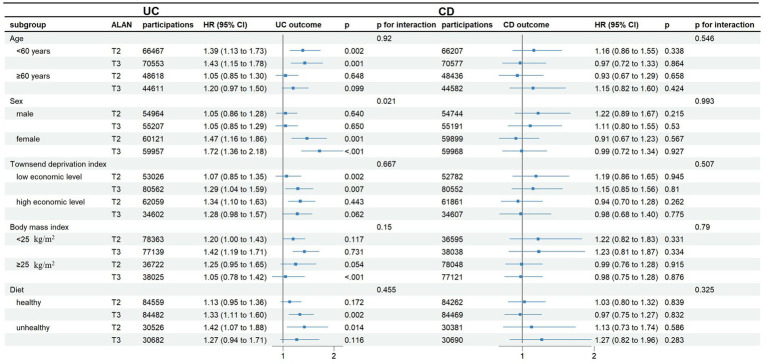
Subgroup analysis for the association between outdoor ALAN and incident IBD according to ALAN tertile. Adjusted for age, sex, race, education attainment, IPAQ, Townsend Deprivation Index, smoking status, alcohol consumption, body mass index, diet, grip strength, waist-to-hip ratio, primary hypertension, type 2 diabetes, hyperthyroidism, hypothyroidism, and mental health status. When the association between each subgroup variable and IBD was evaluated, this variable was excluded from the adjustment. ALAN, artificial light at night; CI, confidence interval; HR, hazard ratio; UC, ulcerative colitis; CD, Crohn’s disease; CI, confidence interval; HR, hazard ratio; UC, ulcerative colitis; CD, Crohn’s disease; IPAQ, International Physical Activity Questionnaire.

Subgroup analysis and interaction tests were performed to examine the association between IBD status and categorical characteristics adjusted in Model 3 (Figure 3). The association between outdoor ALAN and UC incidence appeared stronger in females than in males (*p*-interaction < 0.05). No consistent evidence of potential modifying effects was observed when stratified by age, BMI, economic level and diet subgroups (All *p*-interaction > 0.05).

### 3.5 Sensitivity analysis

In the sensitivity analyses, the results were not materially changed after exclusion of (1) participants who developed IBD within the first 3 years or (2) participants with psychiatric or sleep disorders. Similar results were also observed after additional adjustment for time spent outdoors (summer and winter) and PM_2.5_ (Supplementary Table S1).

## 4 Discussion

In this large prospective UK cohort study with 346,163 adults followed for a median of 13.33 years, the association between outdoor ALAN and incident IBD risk was evaluated. We demonstrated that chronic outdoor ALAN exposure was associated with an increased risk of incident UC but not of CD, and this association remained consistent after adjusting for a wide range of covariates. The exposure–response curve shows a non-linear association between outdoor ALAN exposure and the risk of UC. Additionally, the associations between outdoor ALAN and UC were more pronounced in women than men, and sensitivity analyses confirmed the robustness of the results.

It is widely recognized that the incidence and prognosis of IBD are closely related to environmental exposures (19). A growing body of epidemiological evidence has linked IBD to various environmental factors, including air pollution, residential green/blue space, and microplastics (20–22). Among emerging environmental concerns, ALAN has garnered increasing attention due to its wide-ranging adverse health effects. For instance, night-shift work, a common source of ALAN exposure, has been classified as a Group 2A carcinogen based on evidence of its association with breast, prostate, and colorectal cancers (23, 24). In the previous series of population-based cohort studies, exposure to ALAN led to a series of risk factors for IBD, including impaired glucose homeostasis (25), obesity (26), and sleep disruption (27). Notably, in our analysis, ALAN was a distinct predictor of UC after adjusting for diabetes, sleep duration, and BMI. This finding supports the notion that ALAN may serve as a predictor for UC risk independently of these factors. Another finding of our study is that the association between outdoor ALAN and UC in women was more pronounced in men, which is consistent with the findings of previous studies (28). This may be because women exhibit greater sensitivity to light. At the same light intensity, the suppression of melatonin in women is more pronounced than men (29), which still needs further exploration.

The mechanism linking outdoor ALAN exposure to UC risk is probably multifactorial, primarily mediated through circadian rhythm disruption. Light is the essential input signal for the human circadian system, serving as the key physiological cue that synchronizes the body’s internal rhythms with the external environment (30). ALAN disrupts the natural environmental light cycle, leading to circadian system disruption, which may cause sleep disturbances and suppress pineal melatonin secretion (31). It has been shown that melatonin production in the pineal gland is highly sensitive to light, with even low-intensity, short-duration ALAN exposure inhibiting melatonin secretion (32). In recent years, melatonin has been considered to have great potential in the treatment of UC due to its potent antioxidant properties and its role in protecting the intestinal barrier (33, 34). In a clinical trial involving 30 patients with mild to moderate UC, a 3-month melatonin treatment (3 mg/day) led to significant improvements in the Simple Clinical Colitis Activity Index (SCCAI), fecal calprotectin (FC), and quality of life domains relative to placebo (35). Chen et al. also found that plasma melatonin levels in UC patients were significantly lower than in healthy individuals (36). Taken together, these results suggest that melatonin may play an important role in the pathway between ALAN and UC incidence. In addition, studies have shown that circadian disruption results in heightened immune activation and increased production of pro-inflammatory cytokines (37, 38). After 4 weeks of ALAN exposure, the expression of pro-inflammatory cytokines (TNF and IL-6) in microglia of mice was significantly increased upon LPS stimulation (39). Furthermore, ALAN can disrupt normal gastrointestinal rhythms and cause intestinal dysbiosis, leading to a range of gastrointestinal dysfunctions (40). Modulating the gut microbiota may mitigate the impact of ALAN on IBD. Further work is needed to validate this causal link and assess its clinical applicability.

Notably, outdoor ALAN exposure was not associated with CD incidence in our study, implying that the health effects of outdoor ALAN may vary by subtypes. A potential mechanism is that, *in vitro* studies, melatonin enhances IL-12 secretion in, thereby promoting the Th1 immune response (41, 42), which is thought to be primarily involved in the immune mechanisms of CD rather than UC (43). Interestingly, similar findings have been reported in previous studies (20, 21). More studies are needed to elucidate the underlying mechanisms for different subtypes of IBD.

To the best of our knowledge, this is the first comprehensive prospective cohort study to explore the association between outdoor ALAN exposure and the risk of IBD in a large sample size cohort with detailed information. By adjusting for a range of covariates and performing several sensitivity analyses, we further tested the robustness of our results. Our work offers novel insights into the etiology of IBD and prompts consideration of strategies to mitigate long-term exposure to ALAN. We also have several limitations. Firstly, satellite-measured artificial light pollution may not fully capture individual exposure, as it does not account for exposure from indoor electronic devices, potentially limiting the accuracy of personal exposure assessment. This could lead to exposure misclassification, which would likely attenuate the observed associations. Secondly, since this study is based on population data from the United Kingdom, where the majority of participants are white, the findings may be less generalizable. Also, data for many covariates were obtained through self-reporting, which is subject to potential measurement error or reporting bias. Furthermore, although we conducted sensitivity analyses adjusting for PM₂.₅ and time spent outdoors, we did not account for other environmental exposures, such as residential green and blue space and noise. Future studies should consider these factors. Finally, research has indicated that light at different wavelengths may have distinct effects on health. However, it is not possible to differentiate between these effects in the present study, which will need to be addressed in future research.

## 5 Conclusion

In conclusion, the current study suggests that outdoor ALAN exposure was significantly associated with an increased risk of UC but not CD. Our findings highlight the urgent public health issue of ALAN pollution, and we hope to see increased attention to this matter and anticipate that future measures will be implemented to mitigate the associated harms.

## Data Availability

Publicly available datasets were analyzed in this study. This data can be found at: https://www.ukbiobank.ac.uk/ Project ID:99732.

## Footnotes

1
http://www.r-project.org
