# Supplementary material for: Exposure to outdoor artificial light at night is associated with a higher risk of ulcerative colitis: a prospective cohort study from the UK Biobank
Source: Front Public Health. 2026 Jan 30;14:1704450. doi: 10.3389/fpubh.2026.1704450 (PMC12901493; doi:10.3389/fpubh.2026.1704450)
Supplement: Supplementary file 1 [file Table_1.DOCX]

**Supplementary Materials**

**Exposure To Outdoor Artificial Light At Night is associated with higher risk of Ulcerative Colitis: A Prospective Cohort Study Analysis Of 346,163 Participants In The UK Biobank**

Jiamiao Chen^1,2#^, Laifu Li ^1,2#^, Yan Ran^1,2^, Zhuoya Sun^1,2^, Shiwei Lu^1,2^, Yan Zhuang^1,2^, Lianli Wang^1,2^, Yating Sun^1,2^, Fei Dai^1,2*^

^1^Department of Gastroenterology, The Second Affiliated Hospital of Xi'an Jiaotong University, Xi'an, China

^2^Shaanxi Province Key Laboratory of Gastrointestinal Motility Disorders, Xi'an, China

^#^ Jiamiao Chen and Laifu Li contributed equally to this work.

*Correspondence:

Fei Dai

Email: daifei68@xjtu.edu.cn

Tel: 13772151298

Supplementary Table 1: The results of sensitivity analyses of the association between ALAN and IBD

|  | Sensitivity analysis 1 | | Sensitivity analysis 2 | | Sensitivity analysis 3 | |
| --- | --- | --- | --- | --- | --- | --- |
|  | HR (95%CI) | P | HR (95%CI) | P | HR (95%CI) | P |
| **UC**  ALAN, nW/cm/sr |  |  |  |  |  |  |
| Tercile 1 | ref |  | ref |  | ref |  |
| Tercile 2 | 1.247(1.473,1.056) | 0.009 | 1.215(1.044,1.415) | 0.012 | 1.201(1.026,1.406) | 0.022 |
| Tercile 3 | 1.347(1.599,1.135) | 0.0006 | 1.296(1.108,1.515) | 0.001 | 1.269(1.071,1.505) | 0.006 |
| p for trend |  | <0.0001 |  | <0.0001 |  | <0.0001 |
| **CD**  ALAN, nW/cm/sr |  |  |  |  |  |  |
| Tercile 1 | ref |  | ref |  | ref |  |
| Tercile 2 | 0.988(0.775,1.261) | 0.923 | 1.050(0.842,1.308) | 0.667 | 1.023(0.814,1.285) | 0.847 |
| Tercile 3 | 1.009(0.785,1.298) | 0.942 | 1.025(0.815,1.287) | 0.834 | 0.994(0.774,1.278) | 0.962 |
| p for trend |  | 0.917 |  | 0.721 |  | 0.734 |

Sensitivity analysis 1: exclusion of the participants with psychiatric or sleep disorders.

Sensitivity analysis 2: exclusion of the participants who were diagnosed with IBD within 3 years from baseline.

Sensitivity analysis 3: additionally adjusted for PM 2.5 and time spent in outdoor light as covariates.

HR, hazard ratio; IBD, inflammatory bowel disease; Ref, reference.
